# Evaluation of Intraoperative Retention of Autologous Chondrocytes on Type I/III Collagen Scaffold (Ortho-ACI™) for Cartilage Repair

**DOI:** 10.1186/1757-1146-8-S2-P10

**Published:** 2015-09-22

**Authors:** Raymond Crowe, Craig Willers, Taksum Cheng, Louis Wang, Ming-Hao Zheng

**Affiliations:** 1 Western Hospital, Footscray, VIC; 2 Epworth Hospital, Richmond, VIC; 3 The University of Western Australia, Crawley, WA

**Keywords:** autologous chondrocyte implantation, cartilage repair, type I/III collagen scaffold

## Purpose

To optimise the cost effectiveness of MACI, we have developed a new intraoperative procedure (Ortho-ACI™) of seeding chondrocytes onto the type I/III collagen scaffold and reported on laboratory validation of the procedure and clinical outcomes of Ortho-ACI™.

## Methods and Materials

We tested cell retention on the scaffold by confocal microscopy at 7, 15, 20, 40, 60, 90 and 120 minutes after seeding, and the molecular profile (collagen II, aggrecan, Sox9, HAPLN1) of chondrocytes seeded at 20 minutes and 4 days (preoperative seeding method). Fifteen Ortho-ACI™ patients with 25 cartilage defects were assessed by arthroscopic or magnetic resonance imaging (MRI). Graft repair was graded as excellent, good, poor or no infill. Associations between repair outcome and case variables were also investigated.

## Results

Intraoperative seeded scaffolds had 79% cell retention at 7 minutes, 97% at 20 minutes and 99% at 90 minutes. Molecular profiling at 20 minutes was more consistent with primary chondrocytes than at 4 days. Most grafts were to patella defects (36%), then medial femoral condyle and trochlea (total 80%). Good or excellent MRI outcomes were noted in 100% of grafts at a mean 25 months follow-up (n=5). Good or excellent second look arthroscopy outcomes were noted in 83% of cases at a mean 17 months follow-up (n=24). Graft-related complications were noted in 29% (7/24) of cases, with all presenting as graft edge tissue overgrowth. Six of these 7 overgrowth cases were graded as excellent arthroscopic repair.

## Conclusion

The Ortho-ACI™ procedure, utilizing chondrocytes seeding onto collagen scaffold in the theatre, retains viable cells with suitable molecular profile for implantation. With a limited number of cases, Ortho-ACI™ demonstrated good to excellent MRI and arthroscopic repair outcomes. Although more long-term data is needed, the findings of this study suggest that the Ortho-ACI™ procedure is safe, clinically effective and represents an innovative and cost-effective ACI procedure.

